# Firearms-related injury and sex: a comparative National Trauma Database (NTDB) Study

**DOI:** 10.1136/tsaco-2023-001181

**Published:** 2023-12-12

**Authors:** Catherine Zwemer, Susan Kartiko, Maximilian Peter Forssten, James A Zebley, Joy Dowden Hughes, Babak Sarani, Shahin Mohseni

**Affiliations:** 1 Department of Surgery, The George Washington University School of Medicine and Health Sciences, Washington, District of Columbia, USA; 2 Department of Surgery, The George Washington University Hospital, Washington, District of Columbia, USA; 3 Örebro University School of Medical Sciences, Orebro, Sweden; 4 Department of Orthopedic Surgery, Örebro University Hospital, Örebro, Sweden; 5 Division of Trauma, Critical Care and Acute Care Surgery, Department of Surgery, Sheikh Shakhbout Medical City, Abu Dhabi, UAE; 6 Center for Trauma and Critical Care, The George Washington University Hospital, Washington, District of Columbia, USA; 7 Division of Trauma and Emergency Surgery, Department of Surgery, Sheikh Shakhbout Medical City, Abu Dhabi, UAE

**Keywords:** gender, mortality, Wounds, Gunshot, morbidity

## Abstract

**Background:**

Existing study findings on firearms-related injury patterns are largely skewed towards males, who comprise the majority of this injury population. Given the paucity of existing data for females with these injuries, we aimed to elucidate the demographics, injury patterns, and outcomes of firearms-related injury in females compared with males in the USA.

**Materials and methods:**

A 7-year (2013–2019) retrospective review of the National Trauma Database was conducted to identify all adult patients who suffered firearms-related injuries. Patients who were males were matched (1:1, caliper 0.2) to patients who were females by demographics, comorbidities, injury patterns and severity, and payment method, to compare differences in mortality and several other post-injury outcomes.

**Results:**

There were 196 696 patients admitted after firearms-related injury during the study period. Of these patients, 23 379 (11.9%) were females, 23 378 of whom were successfully matched to a male counterpart. After matching, females had a lower rate of in-hospital mortality (18.6% vs. 20.0%, p<0.001), deep vein thrombosis (1.2% vs. 1.5%, p=0.014), and had a lower incidence of drug or alcohol withdrawal syndrome (0.2% vs. 0.5%, p<0.001) compared with males.

**Conclusion:**

Female victims of firearms-related injuries experience lower rates of mortality and complications compared with males. Further studies are needed to elucidate the cause of these differences.

**Level of evidence:**

Level III.

**WHAT IS ALREADY KNOWN ON THIS TOPIC**

- In the USA, females are 21 times more likely to die from firearm injuries than females in any other developed nations. However, studies conducted using national databases have not compared the outcomes for firearm injuries among females to males.

**WHAT THIS STUDY ADDS**

- Female victims of firearms-related injuries experience lower rates of mortality and complications compared with males.

**HOW THIS STUDY MIGHT AFFECT RESEARCH, PRACTICE OR POLICY**

- Further investigations are required to determine the underlying cause of this relationship, which may aid in the reduction of adverse outcomes for all patients with firearms-related injuries.

## Introduction

Penetrating trauma is the third leading cause of injury in the USA, with more than 218 000 homicides from 2004 to 2018, and accounting for 8% to 10% of all reported injuries.^1–3^ The USA has the highest incidence of firearm fatalities of all developed countries, and firearms-related injuries and mortalities have been rising annually.^2 4 5^

In the USA, females are 21 times more likely to die from firearm injuries than females in any other developed nations,^6^ with previous studies indicating worse outcomes in female patients compared with men.^7 8^ Conversely, small single-institution studies suggest that females are three times less likely to sustain penetrating injuries with less injury severity and half mortality rates compared with male victims.^9–11^

Studies conducted using national databases have not compared the outcomes for firearm injuries among females to males. As the incidence of firearm violence rises, providers should understand the trauma care needed in female versus male victims of firearms-related injuries.^2 4 5^

The objectives of this study were to describe the injury characteristics between the two sexes and determine the differences in outcomes in female victims of firearms-related injuries compared with males.

## Materials and methods

The National Trauma Database (NTDB) was used to identify all adult patients (18 years or older) who suffered a firearms-related injury between 2013 and 2019. Patients were excluded if they had an Abbreviated Injury Scale (AIS) of 6 in any body region, since these injuries are generally not considered survivable, if they were discharged alive in less than 1 day from admission, or if sex was not registered in NTDB. The retrieved data included patient demographics, clinical characteristics, injury severity, comorbidity, and clinical outcomes. This investigation complies with both the Strengthening the Reporting of Observational Studies in Epidemiology guidelines and the Declaration of Helsinki.^12 13^

### Statistical analysis

Patients were divided into two groups based on their sex: female or male. All variables were presented before and after propensity score matching. In the unmatched data, continuous, normally distributed variables were summarized using a mean and SD, with the statistical significance of differences being determined using the Student’s t-test. Continuous, *non-normally* distributed variables were summarized using a median and IQR, with the statistical significance of differences being calculated using the Mann-Whitney U test. Categorical variables were presented as counts and percentages with differences in the distributions being determined using the Χ^2^ test. The primary outcome of interest was in-hospital mortality. Secondary outcomes consisted of hospital length of stay and in-hospital complications.

Confounding was managed using propensity score matching. Male patients were matched to female patients at a 1:1 ratio with a caliper of 0.2. Matching was based on age, race, AIS in each body region, comorbidities (history of myocardial infarction, history of angina, congestive heart failure, hypertension, peripheral vascular disease, cerebrovascular disease, diabetes mellitus, chronic renal failure, dementia, chronic obstructive pulmonary disease, smoking status, liver cirrhosis, coagulopathy, receiving chemotherapy for cancer, metastatic cancer, drug use disorder, alcohol use disorder, and major psychiatric illness), and payment method. After matching, the statistical significance of differences between the cohorts was determined using the paired Student’s t-test (for normally distributed continuous variables), Wilcoxon signed-rank test (for non-normally distributed continuous variables), or McNemar’s test with Bonferroni correction (for categorical variables). Two additional subgroup analyses were also performed. The first analysis only included patients with an Injury Severity Score (ISS) ≥15 to assess differences in the most severely injured patients. The second analysis only included patients who were ≥50 years old to reduce the effect of differing levels of estrogen.

A two-tailed p value of less than 0.05 was considered statistically significant in all analyses. The statistical programming language R (R Foundation for Statistical Computing, Vienna, Austria) was used to perform all calculations and tabulations, using the tidyverse, readxl, writexl, and MatchIt packages.^14^

## Results

During the study period, 196 696 patients who suffered a firearms-related injury met the inclusion and exclusion criteria. This group consisted of 173 317 (88.1%) males and 23 379 (11.9%) females. Prior to propensity score matching, the female cohort were older (females vs males: 32 years vs 29 years, p<0.001), more likely white (45.5% vs 33.3%, p<0.001), more likely to have private insurance (26.0% vs 20.3%, p<0.001), and less likely to be uninsured (23.6% vs 33.4%, p<0.001) (table 1). In general, comorbidities were more prevalent or equally prevalent in female patients compared with their male counterparts (table 2).

**Table 1:** Patient demographics and clinical features among men and women with firearms-related wounds

	Before matching			After matching		
	Male (N=173 317)	Female (N=23 379)	P value	Male (N=23 378)	Female (N=23 378)	P value
Age, median (IQR)	29 (23–39)	32 (24–45)	<0.001	31 (24–44)	32 (24–45)	<0.001
Race, n (%)						
White	57 789 (33.3)	10 631 (45.5)	<0.001	10 540 (45.1)	10 630 (45.5)	0.163
Black	91 396 (52.7)	9886 (42.3)	<0.001	10 062 (43.0)	9886 (42.3)	0.009
Asian	1328 (0.8)	235 (1.0)	<0.001	236 (1.0)	235 (1.0)	1.00
American Indian	1163 (0.7)	223 (1.0)	<0.001	196 (0.8)	223 (1.0)	0.182
Pacific Islander	434 (0.3)	57 (0.2)	0.891	58 (0.2)	57 (0.2)	0.769
Other	16 562 (9.6)	1769 (7.6)	<0.001	1739 (7.4)	1769 (7.6)	0.341
Missing	2863 (1.7)	331 (1.4)		315 (1.3)	331 (1.4)	
ISS, median (IQR)	10 (5.0–19)	10 (5.0–19)	<0.001	10 (5.0–19)	10 (5.0–19)	0.222
Missing, n (%)	4041 (2.3)	384 (1.6)		391 (1.7)	384 (1.6)	
Payment method, n (%)			<0.001			1.00
Private insurance	35 219 (20.3)	6074 (26.0)		6067 (26.0)	6074 (26.0)	
Government insurance	64 331 (37.1)	9978 (42.7)		10 164 (43.5)	9977 (42.7)	
Uninsured	57 915 (33.4)	5506 (23.6)		5417 (23.2)	5506 (23.6)	
Other	8447 (4.9)	893 (3.8)		850 (3.6)	893 (3.8)	
Missing	7405 (4.3)	928 (4.0)		880 (3.8)	928 (4.0)	

Age is measured in years. A patient may have had more than one race.

ISS, Injury Severity Score.

**Table 2:** Comorbidities among males and females with firearms-related wounds

	Before matching			After matching		
	Male (N=173 317)	Female (N=23 379)	P value	Male (N=23 378)	Female (N=23 378)	P value
Hypertension, n (%)	14 337 (8.3)	2473 (10.6)	<0.001	2219 (9.5)	2473 (10.6)	<0.001
History of angina, n (%)	33 (0.0)	4 (0.0)	1.000	4 (0.0)	4 (0.0)	1.00
History of myocardial infarction, n (%)	365 (0.2)	42 (0.2)	0.368	38 (0.2)	42 (0.2)	0.737
Congestive heart failure, n (%)	719 (0.4)	116 (0.5)	0.082	101 (0.4)	116 (0.5)	0.340
History of peripheral vascular disease, n (%)	187 (0.1)	17 (0.1)	0.144	12 (0.1)	17 (0.1)	0.458
Cerebrovascular disease, n (%)	471 (0.3)	92 (0.4)	0.001	76 (0.3)	92 (0.4)	0.247
Diabetes mellitus, n (%)	5756 (3.3)	966 (4.1)	<0.001	900 (3.8)	966 (4.1)	0.109
Chronic renal failure, n (%)	335 (0.2)	20 (0.1)	<0.001	17 (0.1)	20 (0.1)	0.728
Dementia, n (%)	280 (0.2)	52 (0.2)	0.041	45 (0.2)	52 (0.2)	0.542
Chronic obstructive pulmonary disease, n (%)	4174 (2.4)	781 (3.3)	<0.001	694 (3.0)	781 (3.3)	0.016
Bleeding disorder, n (%)	1447 (0.8)	211 (0.9)	0.306	193 (0.8)	211 (0.9)	0.381
Current smoker, n (%)	48 995 (28.3)	5740 (24.6)	<0.001	5492 (23.5)	5739 (24.5)	<0.001
Currently receiving chemotherapy for cancer, n (%)	92 (0.1)	21 (0.1)	0.040	26 (0.1)	21 (0.1)	0.560
Disseminated cancer, n (%)	298 (0.2)	40 (0.2)	1.000	32 (0.1)	40 (0.2)	0.403
Cirrhosis, n (%)	417 (0.2)	41 (0.2)	0.061	35 (0.1)	41 (0.2)	0.561
Advanced directive limiting care, n (%)	607 (0.4)	106 (0.5)	0.016	93 (0.4)	106 (0.5)	0.378

Before propensity score matching, female patients with firearms-related injury were more likely to require intensive care unit (ICU) care (43.5% vs. 42.3%, p<0.001). They also demonstrated similar rates of in-hospital mortality, unplanned admissions to the ICU and pulmonary embolism compared with the unmatched male cohort. However, the rates of surgical site infections (0.7% vs. 0.9%, p<0.001), deep vein thrombosis (DVT) (1.2% vs. 1.6%, p<0.001), and drug or alcohol withdrawal syndrome (0.2% vs. 0.4%, p<0.001) were lower among the female cohort (table 3).

**Table 3:** Patient outcomes among men and women with firearms-related wounds

	Before matching			After matching		
	Male (N=173 317)	Female (N=23 379)	P value	Male (N=23 378)	Female (N=23 378)	P value
Hospital length of stay, median (IQR)	4.0 (2.0–9.0)	4.0 (2.0–9.0)	0.109	4.0 (2.0–9.0)	4.0 (2.0–9.0)	0.058
Missing, n (%)	1970 (1.1)	257 (1.1)		251 (1.1)	257 (1.1)	
Required ICU care, n (%)	73 262 (42.3)	10 173 (43.5)	<0.001	10 332 (44.2)	10 172 (43.5)	0.113
ICU length of stay, median (IQR)	3.0 (2.0–6.0)	3.0 (2.0–6.0)	0.879	3.0 (2.0–7.0)	3.0 (2.0–6.0)	0.020
Required a ventilator, n (%)	52 295 (30.2)	7140 (30.5)	0.254	7583 (32.4)	7139 (30.5)	<0.001
Length of ventilator utilization, median (IQR)	2.0 (1.0–5.0)	2.0 (1.0–5.0)	0.045	2.0 (1.0–5.0)	2.0 (1.0–5.0)	0.042
In-hospital mortality, n (%)	32 000 (18.5)	4359 (18.6)	0.508	4674 (20.0)	4359 (18.6)	<0.001
Unplanned admission to the ICU, n (%)	2292 (1.3)	276 (1.2)	0.078	313 (1.3)	276 (1.2)	0.133
Organ-space surgical site infection, n (%)	1527 (0.9)	156 (0.7)	<0.001	157 (0.7)	156 (0.7)	1.00
Pulmonary embolism, n (%)	1099 (0.6)	129 (0.6)	0.145	138 (0.6)	129 (0.6)	0.622
DVT, n (%)	2719 (1.6)	284 (1.2)	<0.001	346 (1.5)	284 (1.2)	0.014
Drug or alcohol withdrawal syndrome, n (%)	721 (0.4)	52 (0.2)	<0.001	113 (0.5)	52 (0.2)	<0.001

Length of stay is measured in days.

DVT, deep vein thrombosis; ICU, intensive care unit.

After propensity score matching, statistically significant, but clinically non-significant, differences remained in age (32 years vs. 31 years, p<0.001), the proportion of patients who identified as black (42.3% vs. 43.0%, p=0.009), smoking status (24.5% vs. 23.5%, p<0.001), and prevalence of chronic obstructive pulmonary disease (3.3% vs. 3.0%, p=0.016), whereas all other variables were fully balanced (tables 1 and 2 and online supplemental table 1). Females exhibited an overall lower rate of in-hospital mortality (18.6% vs. 20.0%, p<0.001), had a lower incidence of drug or alcohol withdrawal syndrome (0.2% vs. 0.5%, p<0.001), DVTs (1.2% vs. 1.5%, p=0.014), and required mechanical ventilation less frequently than males (30.5% vs. 32.4%, p<0.001) (table 3).

10.1136/tsaco-2023-001181.supp1 Supplementary data

The results remained unchanged in the subgroup analysis consisting of patients with severe firearms-related injury (ISS ≥15). A lower in-hospital mortality rate (44.4% vs. 46.7%, p<0.001), need for mechanical ventilation (59.9% vs. 62.8%, p<0.001), and a lower incidence of drug or alcohol withdrawal syndrome (0.2% vs. 0.6%, p<0.001) were noticed among the female cohort (online supplemental tables 2 and 3).

Similar results were also observed among patients who were ≥50 years old. Compared with male patients, female patients exhibited a lower risk of in-hospital mortality (27.0% vs. 30.2%, p<0.001), a lower need for mechanical ventilation (38.0% vs. 41.5%, p<0.001), and a lower rate of drug or alcohol withdrawal syndrome (0.5% vs. 1.0%, p=0.009) (online supplemental tables 4 and 5).

## Discussion

In this analysis, we aimed to identify the differences in mortality and outcomes after firearms-related injury in the USA between female and male patients admitted to hospitals. We found that females have a statistically significant survival advantage after firearms-related injury compared with males, despite exhibiting a similar overall injury burden after matching. In addition, a lower need for mechanical ventilation and complication rate were detected in the female cohort of patients who had suffered a firearms-related injury.

Although it was not feasible to explain the mechanism for the positive findings associated with the female sex in this retrospective register study, there are several plausible explanations to these findings. The difference in mortality rate between female and male victims of firearms-related injuries may be due to the survival advantage that females have based on their relative hypercoagulable profile compared with males.^15^ Additionally, there may be some advantages in hormonal differences between the two sexes. Estrogen is thought to be protective and immune enhancing, unlike testosterone which has been shown to be immune suppressing.^16–18^ The advantage from estrogen increases with increasing level of bioavailable hormone.^19–21^ Although previous studies have also detected an association between female sex and survival after traumatic insults compared with male sex, this association has been proven to be stronger in premenopausal women regardless of injury mechanism.^9 16 22–25^ Females with firearms-related injuries have also shown to have a lower complication rate compared with males, which could be due to the injury severity-dependent increase of estrogen in the immediate post-traumatic period observed in females.^19^ However, this does not explain why the observed differences remain in patients who are ≥50 years old in the current analysis.

The limitations of our study include the inherent shortcoming of register studies and collection of granular data on care provided. Recording errors may be present due to the dependence on historical records of individual institutions, and medical records have the potential to be incomplete, invalid, or unclear. Propensity score matching in itself is limited as it only controls for recorded/observed variables. As such, any variables not recorded are not accounted for, and should be taken into consideration with analysis of results in this study. Other limitations include a lack of data on patients who died before admission to the trauma center as well as an inability to determine the cause of death in the study population.^17^ On the other hand, this large dataset also allowed for adjustments to be made for a significant amount of pre-admission comorbidities, racial and demographic differences, as well as socioeconomic status, by using payment method as a surrogate.

Future studies are needed to examine these sex disparities in trauma outcomes, and to investigate if differences in levels of trauma care for males and females based on sex-specific physiologic need are needed to provide equitable care to all patients. Despite the limitations, the present study highlights that females admitted to a hospital with firearms-related injury suffer less mortality and experience more favorable outcomes than males in the USA.

## Conclusion

National data of hospitalized trauma patients demonstrate that females have lower mortality and better outcomes than males after admission for firearms-related injury. Further prospective studies are warranted to investigate the ramifications for females and trauma care underlying our findings.

## Data Availability

Data may be obtained from a third party and are not publicly available. The data can be requested from Dr Babak Sarani by the editorial board of *TSACO*.
