# Supplementary material for: Firearms-related injury and sex: a comparative National Trauma Database (NTDB) Study
Source: Trauma Surg Acute Care Open. 2023 Dec 12;8(1):e001181. doi: 10.1136/tsaco-2023-001181 (PMC10753733; doi:10.1136/tsaco-2023-001181)
Supplement: Supplementary data [file tsaco-2023-001181supp001.pdf]

Supplemental Table 1. Additional clinical characteristics among men and women with firearms-related wounds

	Before Matching			After Matching		
	**Male (N = 173,317)**	**Female (N = 23,379)**	**P-value**	**Male (N = 23,378)**	**Female (N = 23,378)**	**P-value**
Head AIS, n (%)			<0.001			1.00
Injury not present	129,942 (75.0)	16,740 (71.6)		16,672 (71.3)	16,739 (71.6)	
1	3,699 (2.1)	394 (1.7)		339 (1.5)	394 (1.7)	
2	1,427 (0.8)	248 (1.1)		251 (1.1)	248 (1.1)	
3	5,250 (3.0)	984 (4.2)		1,022 (4.4)	984 (4.2)	
4	4,611 (2.7)	854 (3.7)		819 (3.5)	854 (3.7)	
5	12,656 (7.3)	2,324 (9.9)		2,376 (10.2)	2,324 (9.9)	
Missing	15,732 (9.1)	1,835 (7.8)		1,899 (8.1)	1,835 (7.8)	
Neck AIS, n (%)			0.015			1.00
Injury not present	148,677 (85.8)	20,248 (86.6)		20,320 (86.9)	20,247 (86.6)	
1	4,390 (2.5)	673 (2.9)		593 (2.5)	673 (2.9)	
2	1,816 (1.0)	260 (1.1)		236 (1.0)	260 (1.1)	
3	1,933 (1.1)	276 (1.2)		226 (1.0)	276 (1.2)	
4	843 (0.5)	104 (0.4)		114 (0.5)	104 (0.4)	
5	152 (0.1)	11 (0.0)		14 (0.1)	11 (0.0)	
Missing	15,506 (8.9)	1,807 (7.7)		1,875 (8.0)	1,807 (7.7)	
Spine AIS, n (%)			0.083			1.00
Injury not present	143,294 (82.7)	19,703 (84.3)		19,868 (85.0)	19,702 (84.3)	
1	66 (0.0)	11 (0.0)		5 (0.0)	11 (0.0)	
2	9,337 (5.4)	1,242 (5.3)		1,072 (4.6)	1,242 (5.3)	
3	1,187 (0.7)	139 (0.6)		132 (0.6)	139 (0.6)	
4	652 (0.4)	82 (0.4)		69 (0.3)	82 (0.4)	
5	3,211 (1.9)	392 (1.7)		350 (1.5)	392 (1.7)	
Missing	15,570 (9.0)	1,810 (7.7)		1,882 (8.1)	1,810 (7.7)	
Thorax AIS, n (%)			<0.001			0.845
Injury not present	110,726 (63.9)	15,494 (66.3)		15,862 (67.9)	15,493 (66.3)	
1	8,137 (4.7)	1,193 (5.1)		1,096 (4.7)	1,193 (5.1)	
2	6,965 (4.0)	979 (4.2)		906 (3.9)	979 (4.2)	
3	19,189 (11.1)	2,525 (10.8)		2,353 (10.1)	2,525 (10.8)	
4	8,509 (4.9)	946 (4.0)		880 (3.8)	946 (4.0)	
5	4,037 (2.3)	422 (1.8)		404 (1.7)	422 (1.8)	
Missing	15,754 (9.1)	1,820 (7.8)		1,877 (8.0)	1,820 (7.8)	

Abdomen AIS, n (%)			<0.001			1.00
Injury not present	105,512 (60.9)	15,524 (66.4)		15,796 (67.6)	15,523 (66.4)	
1	10,857 (6.3)	1,283 (5.5)		1,231 (5.3)	1,283 (5.5)	
2	12,360 (7.1)	1,315 (5.6)		1,231 (5.3)	1,315 (5.6)	
3	15,324 (8.8)	1,824 (7.8)		1,705 (7.3)	1,824 (7.8)	
4	9,753 (5.6)	1,140 (4.9)		1,059 (4.5)	1,140 (4.9)	
5	3,908 (2.3)	485 (2.1)		482 (2.1)	485 (2.1)	
Missing	15,603 (9.0)	1,808 (7.7)		1,874 (8.0)	1,808 (7.7)	
Upper extremity AIS, n (%)			<0.001			1.00
Injury not present	108,790 (62.8)	15,652 (66.9)		15,869 (67.9)	15,652 (67.0)	
1	21,769 (12.6)	2,512 (10.7)		2,355 (10.1)	2,512 (10.7)	
2	19,194 (11.1)	2,385 (10.2)		2,261 (9.7)	2,385 (10.2)	
3	7,988 (4.6)	1,022 (4.4)		1,018 (4.4)	1,022 (4.4)	
4	12 (0.0)	5 (0.0)		3 (0.0)	4 (0.0)	
5	1 (0.0)	0 (0.0)		0 (0.0)	0 (0.0)	
Missing	15,563 (9.0)	1,803 (7.7)		1,872 (8.0)	1,803 (7.7)	
Lower extremity AIS, n (%)			<0.001			1.00
Injury not present	89,191 (51.5)	14,153 (60.5)		13,948 (59.7)	14,152 (60.5)	
1	22,742 (13.1)	2,699 (11.5)		2,772 (11.9)	2,699 (11.5)	
2	11,457 (6.6)	1,313 (5.6)		1,302 (5.6)	1,313 (5.6)	
3	32,061 (18.5)	3,204 (13.7)		3,256 (13.9)	3,204 (13.7)	
4	2,279 (1.3)	201 (0.9)		220 (0.9)	201 (0.9)	
5	53 (0.0)	8 (0.0)		8 (0.0)	8 (0.0)	
Missing	15,534 (9.0)	1,801 (7.7)		1,872 (8.0)	1,801 (7.7)	
External/Other AIS, n (%)			0.185			1.00
Injury not present	151,527 (87.4)	20,797 (89.0)		20,785 (88.9)	20,796 (89.0)	
1	6,325 (3.6)	790 (3.4)		729 (3.1)	790 (3.4)	
2	14 (0.0)	1 (0.0)		2 (0.0)	1 (0.0)	
3	2 (0.0)	0 (0.0)		0 (0.0)	0 (0.0)	
4	2 (0.0)	0 (0.0)		0 (0.0)	0 (0.0)	
5	4 (0.0)	0 (0.0)		0 (0.0)	0 (0.0)	
Missing	15,443 (8.9)	1,791 (7.7)		1,862 (8.0)	1,791 (7.7)	
GCS at admission, n (%)			<0.001			0.597
Mild (GCS 14-15)	124,068 (71.6)	16,451 (70.4)		16,062 (68.7)	16,451 (70.4)	
Moderate (GCS 9-13)	6,888 (4.0)	950 (4.1)		923 (3.9)	949 (4.1)	
Severe (GCS 3-8)	38,353 (22.1)	5,450 (23.3)		5,831 (24.9)	5,450 (23.3)	

Missing	4,008 (2.3)	528 (2.3)		562 (2.4)	528 (2.3)	
Systolic blood pressure, mean (SD)	120 (±44.3)	117 (±41.5)	<0.001	121 (±43.9)	117 (±41.5)	<0.001
Missing, n (%)	6149 (3.5)	834 (3.6)		833 (3.6)	834 (3.6)	
Pulse rate, mean (SD)	87.9 (±34.0)	91.8 (±33.2)	<0.001	87.9 (±33.5)	91.8 (±33.2)	<0.001
Missing, n (%)	4777 (2.8)	637 (2.7)		672 (2.9)	637 (2.7)	
Respiratory rate, mean (SD)	18.4 (±7.5)	18.7 (±7.5)	<0.001	18.1 (±7.5)	18.7 (±7.5)	<0.001
Missing, n (%)	10562 (6.1)	1429 (6.1)		1493 (6.4)	1429 (6.1)	
Temperature, mean (SD)	36.4 (±2.6)	36.4 (±2.5)	0.667	36.4 (±2.5)	36.4 (±2.5)	0.995
Missing, n (%)	43958 (25.4)	5910 (25.3)		5795 (24.8)	5909 (25.3)	
Injury intent, n (%)			<0.001			0.989
Assault	159,525 (72.6)	19,848 (67.0)		19,479 (65.7)	19,848 (67.0)	
Self-inflicted	20,431 (9.3)	4,412 (14.9)		4,414 (14.9)	4,412 (14.9)	
Unintentional	26,861 (12.2)	3,677 (12.4)		4,047 (13.7)	3,677 (12.4)	
Other	4,171 (1.9)	261 (0.9)		562 (1.9)	261 (0.9)	
Undetermined	8,832 (4.0)	1,438 (4.9)		1,134 (3.8)	1,438 (4.9)	
Type of surgery for hemorrhage control, n (%)			<0.001			1.00
None	150,526 (86.9)	20,709 (88.6)		20,726 (88.7)	20,708 (88.6)	
Surgical intervention in the neck	511 (0.3)	59 (0.3)		74 (0.3)	59 (0.3)	
Peripheral vascular surgery	2,784 (1.6)	272 (1.2)		335 (1.4)	272 (1.2)	
Sternotomy	295 (0.2)	34 (0.1)		37 (0.2)	34 (0.1)	
Thoracotomy	4,347 (2.5)	474 (2.0)		492 (2.1)	474 (2.0)	
Laparotomy	13,420 (7.7)	1,596 (6.8)		1,489 (6.4)	1,596 (6.8)	
Surgical procedure performed on a mangled or traumatically amputated extremity	127 (0.1)	10 (0.0)		20 (0.1)	10 (0.0)	
Other surgical intervention	357 (0.2)	59 (0.3)		55 (0.2)	59 (0.3)	
Extraperitoneal pelvic packing	13 (0.0)	3 (0.0)		1 (0.0)	3 (0.0)	
Missing	937 (0.5)	163 (0.7)		149 (0.6)	163 (0.7)	

Supplemental Table 2. Patient demographics and clinical features among men and women with firearms-related wounds and an ISS ≥15, after matching

	Male (N = 8,060)	Female (N = 8,060)	P-value
Age, median [IQR]	32 [24-46]	33 [25-47]	<0.001
Race, n (%)			
White	4,258 (52.8)	4,278 (53.1)	0.467
Black	2,835 (35.2)	2,810 (34.9)	0.331
Asian	86 (1.1)	85 (1.1)	1.00
American Indian	64 (0.8)	68 (0.8)	0.928
Pacific Islander	16 (0.2)	20 (0.2)	0.486
Other	536 (6.7)	539 (6.7)	0.618
Missing	141 (1.7)	144 (1.8)	
ISS, median [IQR]	25 [19-27]	25 [18-27]	0.498
Head AIS, n (%)			1.00
Injury not present	3,837 (47.6)	3,968 (49.2)	
1	58 (0.7)	73 (0.9)	
2	47 (0.6)	49 (0.6)	
3	169 (2.1)	196 (2.4)	
4	843 (10.5)	854 (10.6)	
5	2,439 (30.3)	2,323 (28.8)	
Missing	667 (8.3)	597 (7.4)	
Neck AIS, n (%)			1.00
Injury not present	6,917 (85.8)	6,914 (85.8)	
1	140 (1.7)	176 (2.2)	
2	101 (1.3)	106 (1.3)	
3	157 (1.9)	162 (2.0)	
4	83 (1.0)	104 (1.3)	
5	8 (0.1)	11 (0.1)	
Missing	654 (8.1)	587 (7.3)	
Spine AIS, n (%)			1.00
Injury not present	6,380 (79.2)	6,327 (78.5)	
1	4 (0.0)	3 (0.0)	
2	505 (6.3)	582 (7.2)	
3	76 (0.9)	81 (1.0)	
4	86 (1.1)	82 (1.0)	
5	349 (4.3)	392 (4.9)	
Missing	660 (8.2)	593 (7.4)	
Thorax AIS, n (%)			1.00
Injury not present	4,483 (55.6)	4,348 (53.9)	
1	191 (2.4)	209 (2.6)	
2	298 (3.7)	360 (4.5)	
3	1,115 (13.8)	1,190 (14.8)	
4	924 (11.5)	945 (11.7)	
5	397 (4.9)	420 (5.2)	
Missing	652 (8.1)	588 (7.3)	
Abdomen AIS, n (%)			1.00
Injury not present	4,650 (57.7)	4,629 (57.4)	
1	98 (1.2)	132 (1.6)	
2	243 (3.0)	273 (3.4)	

3	794 (9.9)	816 (10.1)	
4	1,145 (14.2)	1,140 (14.1)	
5	477 (5.9)	485 (6.0)	
Missing	653 (8.1)	585 (7.3)	
Upper extremity AIS, n (%)			1.00
Injury not present	5,964 (74.0)	5,809 (72.1)	
1	691 (8.6)	775 (9.6)	
2	514 (6.4)	610 (7.6)	
3	234 (2.9)	275 (3.4)	
4	3 (0.0)	4 (0.0)	
5	0 (0.0)	0 (0.0)	
Missing	654 (8.1)	587 (7.3)	
Lower extremity AIS, n (%)			1.00
Injury not present	6,143 (76.2)	6,076 (75.4)	
1	376 (4.7)	460 (5.7)	
2	168 (2.1)	143 (1.8)	
3	531 (6.6)	582 (7.2)	
4	182 (2.3)	201 (2.5)	
5	5 (0.1)	8 (0.1)	
Missing	655 (8.1)	590 (7.3)	
External/Other AIS, n (%)			1.00
Injury not present	7,130 (88.5)	7,150 (88.7)	
1	279 (3.5)	326 (4.0)	
2	1 (0.0)	1 (0.0)	
3	0 (0.0)	0 (0.0)	
4	0 (0.0)	0 (0.0)	
5	0 (0.0)	0 (0.0)	
Missing	650 (8.1)	583 (7.2)	
GCS at admission, n (%)			0.985
Mild (GCS 14-15)	3,030 (37.6)	3,186 (39.5)	
Moderate (GCS 9-13)	522 (6.5)	527 (6.5)	
Severe (GCS 3-8)	4,295 (53.3)	4,132 (51.3)	
Missing	213 (2.6)	215 (2.7)	
Systolic blood pressure, mean (SD)	105 (±52.4)	99.9 (±50.3)	<0.001
Missing, n (%)	423 (5.2)	455 (5.6)	
Pulse rate, mean (SD)	86.4 (±42.9)	87.0 (±43.4)	0.348
Missing, n (%)	305 (3.8)	297 (3.7)	
Respiratory rate, mean (SD)	17.0 (±9.5)	17.2 (±9.5)	0.561
Missing, n (%)	805 (10.0)	841 (10.4)	
Temperature, mean (SD)	35.8 (±3.8)	35.9 (±3.1)	0.355
Missing, n (%)	3044 (37.8)	3113 (38.6)	
Type of surgery for hemorrhage control, n (%)			1.00
None	6,201 (76.9)	6,236 (77.4)	
Surgical intervention in the neck	40 (0.5)	40 (0.5)	
Peripheral vascular surgery	114 (1.4)	114 (1.4)	
Sternotomy	39 (0.5)	30 (0.4)	
Thoracotomy	409 (5.1)	382 (4.7)	
Laparotomy	1,122 (13.9)	1,114 (13.8)	
Surgical procedure performed on a mangled or traumatically	2 (0.0)	5 (0.1)	

amputated extremity			
Other surgical intervention	29 (0.4)	31 (0.4)	
Extraperitoneal pelvic packing	1 (0.0)	2 (0.0)	
Missing	103 (1.3)	106 (1.3)	
Payment method, n (%)			1.00
Private insurance	1,962 (24.3)	2,003 (24.9)	
Government insurance	3,180 (39.5)	3,248 (40.3)	
Uninsured	2,215 (27.5)	2,148 (26.7)	
Other	340 (4.2)	329 (4.1)	
Missing	363 (4.5)	332 (4.1)	
Hypertension, n (%)	621 (7.7)	651 (8.1)	0.376
History of angina, n (%)	2 (0.0)	3 (0.0)	1.00
History of myocardial infarction, n (%)	18 (0.2)	14 (0.2)	0.596
Congestive heart failure, n (%)	26 (0.3)	30 (0.4)	0.688
History of peripheral vascular disease, n (%)	3 (0.0)	3 (0.0)	1.00
Cerebrovascular disease, n (%)	35 (0.4)	33 (0.4)	0.902
Diabetes mellitus, n (%)	227 (2.8)	264 (3.3)	0.091
Chronic renal failure, n (%)	5 (0.1)	7 (0.1)	0.752
Dementia, n (%)	18 (0.2)	25 (0.3)	0.360
Chronic obstructive pulmonary disease, n (%)	219 (2.7)	236 (2.9)	0.416
Bleeding disorder, n (%)	65 (0.8)	62 (0.8)	0.856
Current smoker, n (%)	1,225 (15.2)	1,352 (16.8)	0.002
Currently receiving chemotherapy for cancer, n (%)	9 (0.1)	10 (0.1)	1.00
Disseminated cancer, n (%)	19 (0.2)	25 (0.3)	0.440
Cirrhosis, n (%)	8 (0.1)	15 (0.2)	0.190
Advanced directive limiting care, n (%)	64 (0.8)	72 (0.9)	0.530

Age is measured in years. A patient may have had more than one race.

IQR, interquartile range; ISS, injury severity score

Supplemental Table 3. Patient outcomes among men and women with firearms-related wounds and an ISS ≥15, after matching

	**Male (N = 8,060)**	**Female (N = 8,060)**	**P-value**
Hospital length of stay, median [IQR]	6.0 [1.0-14]	6.0 [1.0-14]	0.814
Missing, n (%)	219 (2.7)	214 (2.7)	
Required ICU care, n (%)	5,526 (68.6)	5,440 (67.5)	0.138
ICU length of stay, median [IQR]	4.0 [2.0-8.0]	4.0 [2.0-8.0]	0.628
Required a ventilator, n (%)	5,064 (62.8)	4,824 (59.9)	<0.001
Length of ventilator utilization, median [IQR]	2.0 [1.0-5.0]	2.0 [1.0-5.0]	0.675
In-hospital mortality, n (%)	3,765 (46.7)	3,576 (44.4)	<0.001
Unplanned admission to the ICU, n (%)	190 (2.4)	171 (2.1)	0.338
Organ-space surgical site infection, n (%)	128 (1.6)	99 (1.2)	0.061
Pulmonary embolism, n (%)	89 (1.1)	81 (1.0)	0.585
DVT, n (%)	228 (2.8)	195 (2.4)	0.112
Drug or alcohol withdrawal syndrome, n (%)	47 (0.6)	15 (0.2)	<0.001

Length of stay is measured in days.

ISS, Injury Severity Score; IQR, interquartile range; ICU, intensive care unit

Supplemental Table 4. Patient demographics and clinical features among men and women ≥50 years old with firearms-related wounds, after matching

	Male (N = 4,282)	Female (N = 4,282)	P-value
Age, median [IQR]	57 [53-65]	58 [53-65]	0.605
Race, n (%)			
White	3,010 (70.3)	2,977 (69.5)	0.298
Black	913 (21.3)	902 (21.1)	0.659
Asian	44 (1.0)	48 (1.1)	0.500
American Indian	28 (0.7)	23 (0.5)	0.560
Pacific Islander	4 (0.1)	8 (0.2)	0.386
Other	177 (4.1)	209 (4.9)	0.037
Missing	69 (1.6)	69 (1.6)	
ISS, median [IQR]	10 [5.0-25]	10 [5.0-25]	0.141
Missing, n (%)	60 (1.4)	68 (1.6)	
Head AIS, n (%)			1.00
Injury not present	2,696 (63.0)	2,741 (64.0)	
1	66 (1.5)	74 (1.7)	
2	53 (1.2)	51 (1.2)	
3	217 (5.1)	217 (5.1)	
4	221 (5.2)	216 (5.0)	
5	701 (16.4)	659 (15.4)	
Missing	328 (7.7)	324 (7.6)	
Neck AIS, n (%)			1.00
Injury not present	3,720 (86.9)	3,732 (87.2)	
1	125 (2.9)	118 (2.8)	
2	53 (1.2)	45 (1.1)	
3	45 (1.1)	52 (1.2)	
4	16 (0.4)	16 (0.4)	
5	1 (0.0)	2 (0.0)	
Missing	322 (7.5)	317 (7.4)	
Spine AIS, n (%)			1.00
Injury not present	3,709 (86.6)	3,684 (86.0)	
1	1 (0.0)	2 (0.0)	
2	180 (4.2)	205 (4.8)	
3	21 (0.5)	20 (0.5)	
4	11 (0.3)	9 (0.2)	
5	43 (1.0)	47 (1.1)	
Missing	317 (7.4)	315 (7.4)	
Thorax AIS, n (%)			1.00
Injury not present	2,879 (67.2)	2,829 (66.1)	
1	203 (4.7)	226 (5.3)	
2	204 (4.8)	199 (4.6)	
3	433 (10.1)	471 (11.0)	
4	176 (4.1)	164 (3.8)	
5	65 (1.5)	73 (1.7)	
Missing	322 (7.5)	320 (7.5)	
Abdomen AIS, n (%)			1.00
Injury not present	3,033 (70.8)	2,966 (69.3)	
1	213 (5.0)	206 (4.8)	

2	171 (4.0)	201 (4.7)	
3	290 (6.8)	310 (7.2)	
4	187 (4.4)	202 (4.7)	
5	69 (1.6)	79 (1.8)	
Missing	319 (7.4)	318 (7.4)	
Upper extremity AIS, n (%)			1.00
Injury not present	3,131 (73.1)	3,085 (72.0)	
1	338 (7.9)	376 (8.8)	
2	322 (7.5)	345 (8.1)	
3	173 (4.0)	158 (3.7)	
4	0 (0.0)	1 (0.0)	
5	0 (0.0)	0 (0.0)	
Missing	318 (7.4)	317 (7.4)	
Lower extremity AIS, n (%)			1.00
Injury not present	2,851 (66.6)	2,896 (67.6)	
1	435 (10.2)	413 (9.6)	
2	185 (4.3)	192 (4.5)	
3	467 (10.9)	434 (10.1)	
4	25 (0.6)	28 (0.7)	
5	2 (0.0)	3 (0.1)	
Missing	317 (7.4)	316 (7.4)	
External/Other AIS, n (%)			1.00
Injury not present	3,860 (90.1)	3,833 (89.5)	
1	103 (2.4)	133 (3.1)	
2	2 (0.0)	1 (0.0)	
3	0 (0.0)	0 (0.0)	
4	0 (0.0)	0 (0.0)	
5	0 (0.0)	0 (0.0)	
Missing	317 (7.4)	315 (7.4)	
GCS at admission, n (%)			1.00
Mild (GCS 14-15)	2,515 (58.7)	2,635 (61.5)	
Moderate (GCS 9-13)	179 (4.2)	177 (4.1)	
Severe (GCS 3-8)	1,481 (34.6)	1,371 (32.0)	
Missing	107 (2.5)	99 (2.3)	
Systolic blood pressure, mean (SD)	118 (±49.4)	119 (±47.2)	0.250
Missing, n (%)	195 (4.6)	180 (4.2)	
Pulse rate, mean (SD)	83.5 (±34.5)	85.6 (±32.4)	0.010
Missing, n (%)	146 (3.4)	131 (3.1)	
Respiratory rate, mean (SD)	17.4 (±8.0)	18.0 (±7.5)	0.002
Missing, n (%)	369 (8.6)	337 (7.9)	
Temperature, mean (SD)	36.1 (±2.9)	36.2 (±2.7)	0.757
Missing, n (%)	1198 (28.0)	1171 (27.3)	
Type of surgery for hemorrhage control, n (%)			1.00
None	3,753 (87.6)	3,793 (88.6)	
Surgical intervention in the neck	19 (0.4)	15 (0.4)	
Peripheral vascular surgery	53 (1.2)	41 (1.0)	
Sternotomy	11 (0.3)	8 (0.2)	
Thoracotomy	82 (1.9)	66 (1.5)	
Laparotomy	302 (7.1)	315 (7.4)	
Surgical procedure performed	4 (0.1)	0 (0.0)	

on a mangled or traumatically amputated extremity			
Other surgical intervention	21 (0.5)	12 (0.3)	
Extraperitoneal pelvic packing	1 (0.0)	0 (0.0)	
Missing	36 (0.8)	32 (0.7)	
Payment method, n (%)			1.00
Private insurance	1,153 (26.9)	1,189 (27.8)	
Government insurance	2,079 (48.6)	2,063 (48.2)	
Uninsured	734 (17.1)	727 (17.0)	
Other	153 (3.6)	144 (3.4)	
Missing	163 (3.8)	159 (3.7)	
Hypertension, n (%)	1,286 (30.0)	1,334 (31.2)	0.228
History of angina, n (%)	3 (0.1)	3 (0.1)	1.00
History of myocardial infarction, n (%)	34 (0.8)	27 (0.6)	0.435
Congestive heart failure, n (%)	81 (1.9)	75 (1.8)	0.687
History of peripheral vascular disease, n (%)	10 (0.2)	10 (0.2)	1.00
Cerebrovascular disease, n (%)	39 (0.9)	56 (1.3)	0.097
Diabetes mellitus, n (%)	436 (10.2)	497 (11.6)	0.026
Chronic renal failure, n (%)	10 (0.2)	12 (0.3)	0.823
Dementia, n (%)	42 (1.0)	48 (1.1)	0.590
Chronic obstructive pulmonary disease, n (%)	292 (6.8)	315 (7.4)	0.340
Bleeding disorder, n (%)	62 (1.4)	70 (1.6)	0.536
Current smoker, n (%)	830 (19.4)	820 (19.1)	0.789
Currently receiving chemotherapy for cancer, n (%)	8 (0.2)	12 (0.3)	0.502
Disseminated cancer, n (%)	32 (0.7)	34 (0.8)	0.899
Cirrhosis, n (%)	25 (0.6)	23 (0.5)	0.883
Advanced directive limiting care, n (%)	81 (1.9)	75 (1.8)	0.675

Age is measured in years. A patient may have had more than one race.

IQR, interquartile range; ISS, injury severity score

Supplemental Table 5. Patient outcomes among men and women ≥50 years old with firearms-related wounds, after matching

	**Male (N = 4,282)**	**Female (N = 4,282)**	**P-value**
Hospital length of stay, median [IQR]	4.0 [2.0-10]	4.0 [2.0-10]	0.079
Missing, n (%)	79 (1.8)	66 (1.5)	
Required ICU care, n (%)	2,117 (49.4)	2,142 (50.0)	0.586
ICU length of stay, median [IQR]	3.0 [2.0-8.0]	3.0 [2.0-7.0]	0.148
Missing, n (%)	2165 (50.6)	2140 (50.0)	
Required a ventilator, n (%)	1,778 (41.5)	1,626 (38.0)	<0.001
Length of ventilator utilization, median [IQR]	2.0 [1.0-5.0]	2.0 [1.0-5.0]	0.753
Missing, n (%)	2504 (58.5)	2656 (62.0)	
In-hospital mortality, n (%)	1,292 (30.2)	1,158 (27.0)	<0.001
Unplanned admission to the ICU, n (%)	90 (2.1)	75 (1.8)	0.264
Organ-space surgical site infection, n (%)	38 (0.9)	31 (0.7)	0.470
Pulmonary embolism, n (%)	40 (0.9)	30 (0.7)	0.282
DVT, n (%)	82 (1.9)	59 (1.4)	0.060
Drug or alcohol withdrawal syndrome, n (%)	43 (1.0)	21 (0.5)	0.009

Length of stay is measured in days.

ISS, Injury Severity Score; IQR, interquartile range; ICU, intensive care unit
